# Brain Lipopolysaccharide Preconditioning-Induced Gene Reprogramming Mediates a Tolerance State in Electroconvulsive Shock Model of Epilepsy

**DOI:** 10.3389/fphar.2018.00416

**Published:** 2018-05-01

**Authors:** Elham Amini, Mojtaba Golpich, Abdoreza S. Farjam, Behnam Kamalidehghan, Zahurin Mohamed, Norlinah M. Ibrahim, Abolhassan Ahmadiani, Azman A. Raymond

**Affiliations:** ^1^Department of Medicine, Faculty of Medicine, University Kebangsaan Malaysia Medical Centre, National University of Malaysia, Kuala Lumpur, Malaysia; ^2^Institute of Tropical Agriculture and Food Security, Universiti Putra Malaysia, Selangor, Malaysia; ^3^Department of Medical Genetics, School of Medicine, Shahid Beheshti University of Medical Sciences, Tehran, Iran; ^4^Department of Pharmacology, Faculty of Medicine, University of Malaya, Kuala Lumpur, Malaysia; ^5^Neuroscience Research Center, Shahid Beheshti University of Medical Sciences, Tehran, Iran

**Keywords:** seizures, neuroprotection, preconditioning, tolerance, treatment, gene reprogramming, brain injury, signaling pathway

## Abstract

There is increasing evidence pointing toward the role of inflammatory processes in epileptic seizures, and reciprocally, prolonged seizures induce more inflammation in the brain. In this regard, effective strategies to control epilepsy resulting from neuroinflammation could be targeted. Based on the available data, preconditioning (PC) with low dose lipopolysaccharide (LPS) through the regulation of the TLR4 signaling pathway provides neuroprotection against subsequent challenge with injury in the brain. To test this, we examined the effects of a single and chronic brain LPS PC, which is expected to lead to reduction of inflammation against epileptic seizures induced by electroconvulsive shock (ECS). A total of 60 male Sprague Dawley rats were randomly assigned to five groups: control, vehicle (single and chronic), and LPS PC (single and chronic). We first recorded the data regarding the behavioral and histological changes. We further investigated the alterations of gene and protein expression of important mediators in relation to TLR4 and inflammatory signaling pathways. Interestingly, significant increased presence of NFκB inhibitors [Src homology 2-containing inositol phosphatase-1 (SHIP1) and Toll interacting protein (TOLLIP)] was observed in LPS-preconditioned animals. This result was also associated with over-expression of IRF3 activity and anti-inflammatory markers, along with down-regulation of pro-inflammatory mediators. Summarizing, the analysis revealed that PC with LPS prior to seizure induction may have a neuroprotective effect possibly by reprogramming the signaling response to injury.

## Introduction

A series of complex events are believed to trigger subsequent changes in the brain causing epilepsy. While the underlying cause of epilepsy remains largely unknown, increasing evidence supports links between neuroinflammation and epilepsy (Dupuis and Auvin, 2015; Vezzani et al., 2016). Several studies have highlighted that neuroinflammation is associated with an increased production of pro-inflammatory cytokines which normally contribute to neuronal damage and other events that make structural and functional changes in the hippocampus to initiate epileptogenesis (Iori et al., 2013; Amini et al., 2015; Kosonowska et al., 2015). Moreover, inflammatory processes in cell injury resulting from prolonged seizures also trigger molecular and cellular alterations, which leads to the production of vast numbers of different pro-inflammatory mediators (Amhaoul et al., 2015; Vezzani et al., 2016). So, in this regard, it is believed that neuroinflammation can be both a consequence and also a cause of epileptic seizures (Amhaoul et al., 2015; Gershen et al., 2015).

Previous experimental work, which has been conducted in animal models suggested that induction of seizures elicited by either various chemical agents (Kaur et al., 2014; Kołosowska et al., 2014) or repeated electrical stimulation such as ECS (Cardoso et al., 2011; Rothan et al., 2017) will ultimately lead to progressive development of seizures. Despite the development of new treatment approaches that have made great advances in the control of seizures, intolerable side effects of currently available treatments still pose a significant problem (Amhaoul et al., 2014; Moshé et al., 2015). Therefore, it is necessary to seek and explore novel and potential treatments for prevention of seizures in patients with epilepsy.

Recently, insights into neuroprotective mechanisms of the brain to modulate noxious stimuli and then recover itself from injury have opened up new prospects for the development of new and effective therapies. From this perspective, it is believed that PC by mild persisting inflammation may pave the way to novel treatments to modulate neurological disorders (Dmowska et al., 2010; Mirrione et al., 2010; Hickey et al., 2011; Vartanian et al., 2011). PC with a sub-lethal dose of LPS is one of the best characterized neuroprotective stimuli for inducing mild inflammation in the rodent brain (Hickey et al., 2011; Wang et al., 2015). Low doses of LPS can alter the genomic response by a process of reprogramming, which occurs through the TLR4 signaling pathway, mainly by activation of TRIF-dependent cascade and the inhibition of the MYD88-dependent pathway (Wang et al., 2015). This leads to a neuroprotective response through activation of anti-inflammatory markers (e.g., IFN-β and IL10) by increasing IRF3 and also inducing NFκB inhibitors (SHIP1 and TOLLIP) that are crucial to reduce NFκB production (Vartanian et al., 2011). Therefore, it ultimately reduces the generation of pro-inflammatory cytokines (e.g., TNF-α and IL-1β), modulates the inflammatory responses, and protects the brain against subsequent serious injury (Schaafsma et al., 2015).

Given the evidence above, we hypothesized that a single low dose and chronic ultra-low dose of brain LPS PC may have beneficial effects on neuronal survival following epileptic injuries by repeated seizures in the ECS model of epilepsy. Therefore, behavioral impairments related to epileptic seizures, lesions in hippocampal regions (CA1, CA3, and DG), and the molecular mechanisms underlying TLR4 and inflammatory signaling pathways were evaluated.

## Materials and Methods

### Animals

**FIGURE 1:**
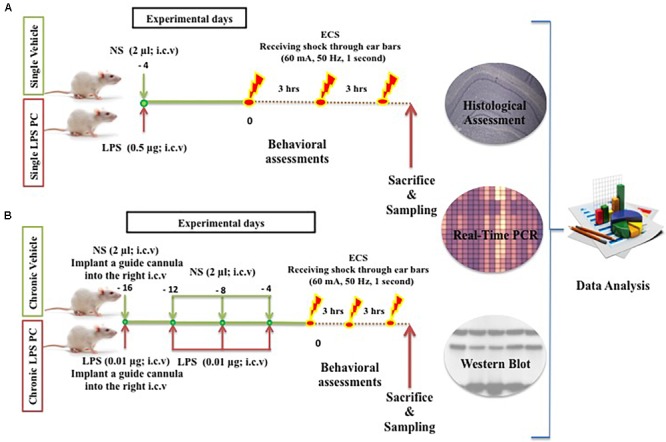
Schematic diagram of experimental design and procedures in the **(A)** single and **(B)** chronic LPS preconditioning and vehicle groups in this study.

Adult male SD rats (200–250 g) were used and these were obtained from the Universiti Kebangsaan Malaysia (UKM) Vendor (Kuala Lumpur, Malaysia). The handling of animals was carried out according to the National Institutes of Health guidelines and regulations. The animals were housed in a cage under controlled environmental conditions for temperature (∼24°C), humidity (∼50%), and light (a daily ratio of 1:1). The animals were allowed access to food and water *ad libitum* (free access to food and water). All efforts were made to minimize animal suffering and the number of animals required. The procedures involving animals were approved by the Animal Ethics Committee, Faculty of Medicine, UKM (Ethics No: FP/MED/NORLINAH/31-JAN-/493-FEB-2013-FEB-2016). The experimental designs, dosage, interval, and frequency of drugs administration are illustrated below (**Figure 1**).

### Surgical Procedures

In this experiment, a total of 60 male SD rats were randomly assigned to five groups, namely, control (*n* = 12), single vehicle (*n* = 12), chronic vehicle (*n* = 12), single LPS PC (*n* = 12), and chronic LPS PC groups (*n* = 12). Animals allocated for LPS PC and matched vehicle groups were randomized and each rat was i.c.v injected with either LPS (*Escherichia coli 055:B5*) or an equivalent volume of NS. In the single LPS preconditioned group, animals received one injection of low dose LPS at 4 days prior to receiving shock, while in the chronic preconditioned group, animals received four injections of ultra-low doses LPS (one injection every 4 days), initiated from 16 days prior to receiving shock. Rats in the vehicles groups were pretreated with NS.

Rats were deeply anesthetized with ketamine, xylazine (0.1 ml/100 gm BW; i.p) and then placed on a stereotaxic surgery apparatus (David KOPF Instruments, United States). Injection of LPS or NS into the i.c.v of preconditioned and vehicle animals was performed under coordination of (relative to bregma): AP: -0.96 mm (posterior to bregma), ML: 2.00 mm (lateral to the midline of the skull), and DV: -3.8 mm (ventral to the dura surface), based on the atlas of Paxinos and Watson (1998). A Hamilton syringe needle was used for LPS injection at a dose of 0.5 μg (0.25 μg/μl) for single injection and 0.01 μg (0.005 μg/μl) for chronic injections (every 4 days, for four injections totally). The solution was delivered at a rate of 2 μl over 2 min. The needle remained in place for three more minutes post injection to avoid reflux through the path of the needle. Upon removal, a stainless steel guide cannula was implanted into the right i.c.v for microinjection. Finally, the guide cannula was fixed and the wound was closed with dental cement. Aseptic conditions prevailed for all surgical procedures performed. After surgery, rats were allowed to recover in new cages.

### Induction of Seizures and Behavioral Assessments

In the ECS group, rats received a course of three ECS seizures in total, administered at 3-h intervals (i.e., at 0, 3, and 6 h). Each stimulus (50 Hz, 60 mA for 1 s) was delivered via ear-clip electrodes wired to a stimulus generator. Animals were behaviorally observed to ensure that full tonic–clonic seizures occurred. The seizure duration was recorded for all animals. In the control groups, animals were handled identically to the ECS-treated rats, and contact was made with the electrodes, except that no current was passed through the electrodes. All animals in this model were observed for 1 h after ECS administration. Animals were also excluded if they displayed any evidence of tonic–clonic seizures with hind limb involvement.

### Tissue Preparation and Histology

Following the completion of the behavioral experiments, all animals were sacrificed by decapitation using a guillotine. Subsequently, the brains were quickly removed and dissected on ice. For the molecular assays such as gene and protein analysis, the right half of the hippocampus was dissected quickly, frozen in liquid nitrogen, and then stored at -80°C. In order to perform the histological assessment, the left half of the brains was immediately cut according to the atlas by Paxinos and Watson (1998). It was then fixed with a fixative solution containing 4% freshly prepared paraformaldehyde (Sigma, United States) in pH 7.4, at 4°C at least for 10 days.

Upon fixation, brain samples were routinely embedded in paraffin wax (Leica) and placed on embedding blocks. Three tissue blocks were selected randomly. The selected embedded tissue blocks were sectioned (5 μm thickness) using a microtome rotary (Leica), placed on glass slides, and stained with cresyl violet (Nissl Staining, Sigma). The CA1, CA3, and DG regions were evaluated under a light microscope (Nikon) and captured at fields of 400× magnification at the same locations within the hippocampal regions. The cells with a well-defined nucleolus and typical Nissl bodies were counted with the ImageJ 1.50 b software using the cell counting function. To avoid any bias in cell counting, this procedure was performed blind by an experienced independent observer.

### RNA Isolation and Gene Expression

To evaluate the effective role of LPS PC as protection following tonic–clonic seizures, the expression level of important genes under TLR4 and inflammation pathways was examined. Frozen hippocampal tissues stored at -80°C were selected randomly by an investigator blinded to the treatment groups. Extraction of total RNA from the hippocampus was performed using RNeasy Plus Universal Mini kit (Qiagen Inc, Valencia, CA, United States) according to the instruction manual. Then, RNA quality and quantity were determined with nanodrop UV/V spectrophotometry, native agarose gel, and Agilent 2100 Bioanalyzer (Agilent Technologies, Inc., California), in accordance with the manufacturers’ protocols. Following this, RNA was reverse transcribed to cDNA using high-capacity cDNA reverse transcription kits (Applied Biosystem). PCR reactions were finally carried out using a Biomark HD System (Fluidigm, South San Francisco, United States). The Fluidigm system with an IFC chip is a new platform for high-throughput RT-PCR. It is a reliable and sensitive method for measuring RNA transcript levels and performs quantification of multiple RNA targets simultaneously in nanoliter volumes. The expression level of 11 selected genes was evaluated using the IFC chips available for dynamic analysis (192 samples × 24 assays). In this assay, three replicates per each sample were tested. The expression of all genes was normalized to the housekeeping gene GAPDH relative to the expression level. The sequences of all primers are listed in **Table 1**.

**Table 1:** List of primer sequences used for RT-PCR analysis.

Primer	Forward	Reverse
GAPDH	GGGTGCCCAGTACAGTAGGA	ATTCCCAACCAACAGTGAGC
TLR4	ATGCCAGTGCTTGTGTGGTA	ACCATCCACCTATCCATCCA
SHIP1	AAGCCCGAGATGTTTGAGAA	CATGATGCTGGGTGAAGAGA
TOLLIP	AGGATGGAAGGAAGGAAGGA	CAAGTTGCCAAGCAATCTCA
NFκB	TCTCCCACACAGTGACAGGA	TCTCGCTGTGTGTGTTCCTC
IRF3	CCTGTATGTCAAGGGGCAAA	TGGAAAACTGTGGGGTAGGA
TNF-α	TGACCCCCATTACTCTGACC	TTCAGCGTCTCGTGTGTTTC
IL1-β	TGTTTGAGCAGCAAGGACAC	ACTAGGCGTACATGGCAACC
IFN-β	CAGCTACAGGACGGACTTCA	AGTCTCATTCCACCCAGTGC
IL10	CTTCCTTCTGCCTGTGAACC	TGCGTGTGTAGGCAGTCTTC
NR_2_B	TCCTTTGCCAACAAGTCCTC	TGAAGCAAGCACTGGTCATC
GABA_A_	CACATGGAGGAAGGGGACTA	GAGGTCCTCCACACTTCTGC

### Protein Assay

Brain samples were selected randomly by an investigator blinded to the treatment groups, homogenized in a Potter Elvehjem tissue grinder (Sigma, St Louis, MO, United States) using 1 ml chilled Tris buffer (20 mM Tris, pH 7.5; 0.75 M NaCl; 2 mM 2-mercaptoethanol) with 10 μl/ml protease inhibitor cocktail (lot no. P8340, Sigma, St Louis, MO, United States), and centrifuged at 23,000*g* for 45 min at 4°C. The protein concentrations of the supernatants were quantified using a bicinchoninic acid protein assay kit (BCA-1, B9643, Sigma–Aldrich, St Louis, MO, United States) with BSA as the standard. Total protein (25 μg) was loaded and separated on 10% polyacrylamide gels containing sodium dodecyl sulfate using a mini gel apparatus. Gels were electrophoresed at 120 V until the tracking dye reached the base of the gel. The fractionated proteins were transferred to polyvinylidene difluoride membranes (MSI, Westborough, MA, United States) using a semidry electrophoretic transfer cell. The non-specific binding sites were blocked by 10 ml cold blocking buffer TBST + BSA for 60 min. The membranes were incubated for 1 h with 5 ml blocking buffer containing antiserum monoclonal antibodies for SHIP1 (Santa Cruz), IRF3 (Santa Cruz), and NFκB (Abcam) and polyclonal antibodies for TOLLIP (Abcam) and β-actin (Sigma) in a 1:1000 dilution. Following 1 h of incubation, the blots were washed three times (5 min each) with 10 ml cold TBST. Blots were then incubated with horseradish-peroxidase-conjugated anti-mouse (Abcam) or antibody-rabbit (Abcam) secondary antibodies for 30 min in a 1:2000 dilution. After rinsing with cold TBST (three times, 5 min each), the blots were exposed to an enhanced chemiluminescent substrate (ChemiGlow; Alpha Innotech, San Leandro, CA, United States). Visualization of bands was performed using a chemiluminescent imaging system (FluorChem 5500; Alpha Innotech) followed by quantification of the band summation density by the ImageJ 1.50b software. Sizes of the immunodetected proteins were confirmed by molecular weight markers (Precision Plus Protein, Bio-Rad, Hercules, CA, United States). All solutions were made with Milli-Q water (Millipore, Bedford, MA, United States).

### Statistical Analysis

All data were analyzed using the Student’s *t*-test and one-way ANOVA procedure of GraphPad Prism and means were separated using Tukey *post hoc* test. In all analyses, ρ < 0.05 was considered statistically significant. All data were presented as mean ± SEM.

## Results

### Effects of LPS Preconditioning on Behavioral Assessments

**FIGURE 2:**
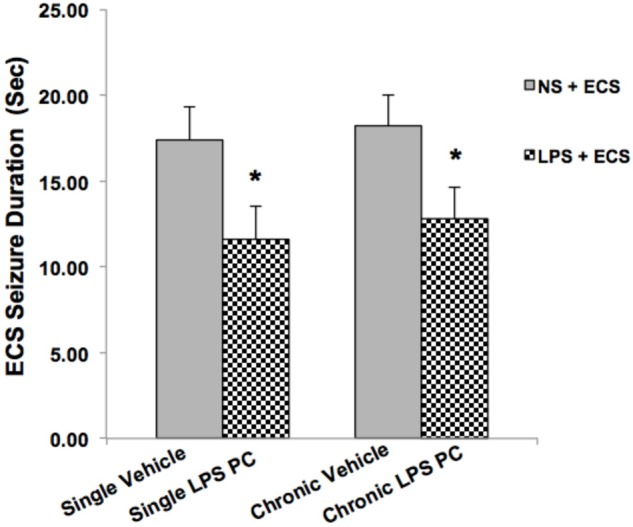
Effect of preconditioning by single low dose and chronic ultra-low dose of LPS on seizure duration induced by ECS administration in a rat model. Asterisk (^∗^) denotes a significant difference between the preconditioned groups and vehicle groups. Statistical analysis was done according to Student’s *t*-test with significance levels of ^∗^ρ < 0.05 vs. the vehicle groups. Data are shown as mean ± SEM of 12 animals (*n* = 12). ECS, electroconvulsive shock; NS, normal saline; LPS, lipopolysaccharide; PC, preconditioning.

Animals in the ECS group (except control groups) received a series of three ECSs delivered via ear clip electrodes using a pulse generator in a day. Animals were behaviorally observed to ensure that tonic–clonic seizures occurred. Data analysis of behavioral assessments was done only on the vehicle groups since there were no tonic–clonic seizures recorded for the control animals in this model. Our observations indicated significant reduction in the duration of seizures induced by ECS in single and chronic preconditioned groups compared to the vehicle animals (**Figure 2**). In our study, all behavioral changes including duration of tonic–clonic seizures, latency, and mortality induced by ECS were recorded. As all animals in the ECS model were evoked immediately after receiving shock, the seizure latency was not observed in this model. (The latency was defined as the time of onset of the first tonic–clonic seizures in ECS-induced animals.) Although, during our behavioral observation, administration of ECS exhibited high scores of tonic–clonic seizures, there was no mortality in the ECS-treated groups.

### The Effect of LPS Preconditioning on the Cell Density in the Regions of the Hippocampus

**FIGURE 3:**
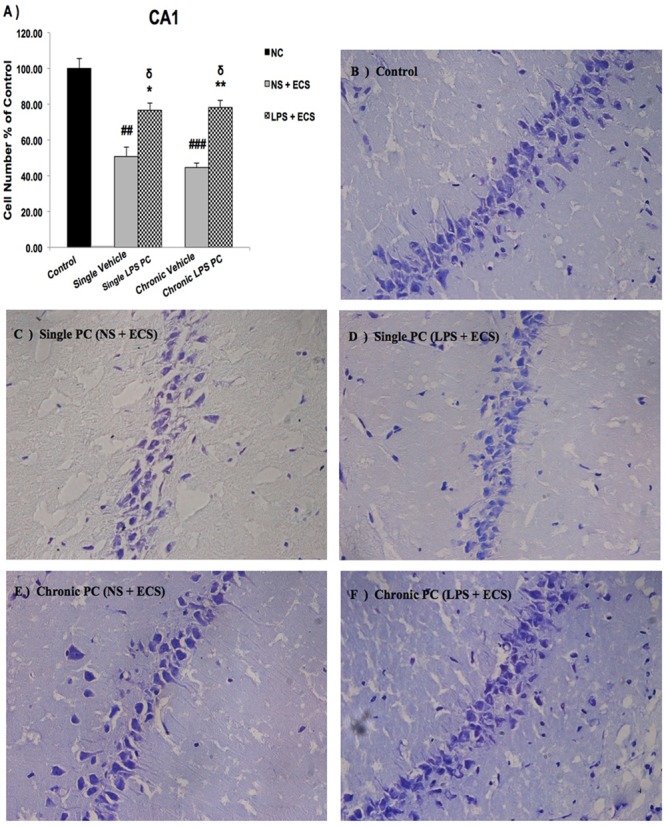
Photomicrographs of Nissl-stained CA1 sub-region of rat brain hippocampus in the ECS model of epilepsy. **(A)** Effect of single low dose and chronic ultra-low dose of LPS preconditioning on the number of CA1 cells, **(B)** control, **(C)** vehicle (single NS), **(D)** single LPS preconditioned groups, **(E)** vehicle (chronic NS), and **(F)** chronic LPS preconditioned groups in the ECS model. Scale bars are equal to 50 μm. Asterisk (^∗^) denotes a significant difference between the preconditioned groups and vehicle groups. Delta (δ) denotes a significant difference between the preconditioned groups and control groups. Hash (#) denotes a significant difference between the vehicle groups and control groups. Statistical analysis has been done according to the one-way ANOVA with significance levels of ^∗^ρ < 0.05 and ^∗∗^ρ < 0.01; ^δ^ρ < 0.05; ^##^ρ < 0.01, and ^###^ρ < 0.001. Data are shown as mean% ± SEM of three animals (*n* = 3), which expressed as the cell number percentage with respect to the control. ECS, electroconvulsive shock; NS, normal saline; LPS, lipopolysaccharide; PC, preconditioning; NC, no current was passed through the electrodes.

**FIGURE 4:**
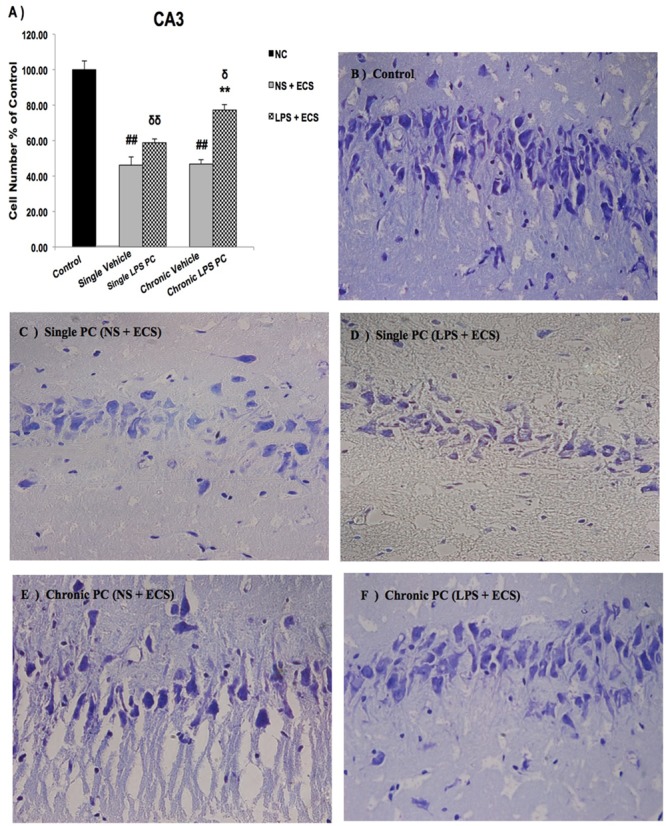
Photomicrographs of Nissl-stained CA3 sub-region of rat brain hippocampus in the ECS model of epilepsy (*n* = 3). **(A)** Effect of single low dose and chronic ultra-low dose of LPS preconditioning on the number of CA3 cells, **(B)** control, **(C)** vehicle (single NS), **(D)** single LPS preconditioned groups, **(E)** vehicle (chronic NS), and **(F)** chronic LPS preconditioned groups in the ECS model. Scale bars are equal to 50 μm. Asterisk (^∗^) denotes a significant difference between the preconditioned groups and vehicle groups. Delta (δ) denotes a significant difference between the preconditioned groups and control groups. Hash (#) denotes a significant difference between the vehicle groups and control groups. Statistical analysis has been done according to the one-way ANOVA with significance levels of ^∗∗^ρ < 0.01; ^δ^ρ < 0.05, ^δδ^ρ < 0.01; and ^##^ρ < 0.01. Data are shown as mean% ± SEM of three animals (*n* = 3), which expressed as the cell number percentage with respect to the control. ECS, electroconvulsive shock; NS, normal saline; LPS, lipopolysaccharide; PC, preconditioning; NC, no current was passed through the electrodes.

**FIGURE 5:**
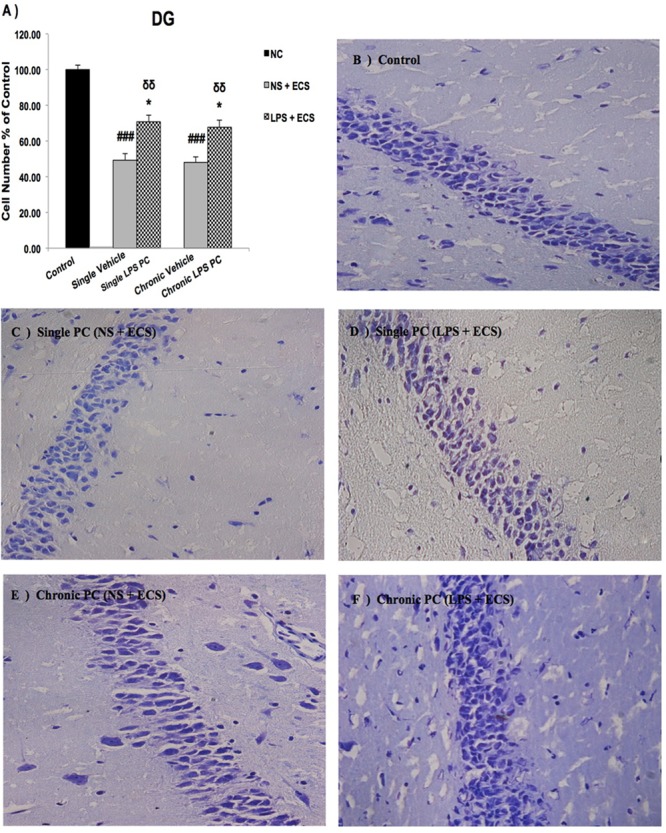
Photomicrographs of Nissl-stained DG sub-region of rat brain hippocampus in the ECS model of epilepsy (*n* = 3). **(A)** Effect of single low dose and chronic ultra-low dose of LPS preconditioning on the number of DG cells, **(B)** control, **(C)** vehicle (single NS), **(D)** single LPS preconditioned groups, **(E)** vehicle (chronic NS), and **(F)** chronic LPS preconditioned groups in the ECS model. Scale bars are equal to 50 μm. Asterisk (^∗^) denotes a significant difference between the preconditioned groups and vehicle groups. Delta (δ) denotes a significant difference between the preconditioned groups and control groups. Hash (#) denotes a significant difference between the vehicle groups and control groups. Statistical analysis has been done according to the one-way ANOVA with significance levels of ^∗^ρ < 0.05; ^δδ^ρ < 0.01; and ^###^ρ < 0.001. Data are shown as mean% ± SEM of three animals (*n* = 3), which expressed as the cell number percentage with respect to the control. ECS, electroconvulsive shock; NS, normal saline; LPS, lipopolysaccharide; PC, preconditioning; NC, no current was passed through the electrodes.

We analyzed the severity of cell death in the regions of the hippocampus. The data presented in **Figures 3–5** demonstrate a remarkable decrease in cell number in the CA1, CA3, and DG in the vehicle groups compared with the control groups. The damaged neurons were characterized by nuclear fragments and shrunken cell bodies. In this part of the study, the cell percentage was expressed as mean% ± SEM of three animals (*n* = 3). However, animals pretreated with LPS demonstrated resistance to neurodegeneration and attenuation of neuronal loss.

Our results showed a significant increase in the percentage of cell survival in the CA1 region in both single and chronic preconditioned groups compared with the vehicle groups (**Figure 3**). Moreover, in the CA3 region, chronic LPS PC significantly reduced the neuronal degeneration compared with the vehicle groups as reflected by the significantly higher percentage of cell survival. In contrast, in the single LPS PC group, no significant change in the percentage of cell survival was observed (**Figure 4**). In the DG region, single and chronic LPS PC significantly reduced the neuronal damage compared with the vehicle groups with higher percentage of cell survival (**Figure 5**).

### Effect of LPS Preconditioning on TLR4 Signaling Pathway

To elucidate possible mechanisms of neuroprotection by low dose and ultra-low doses of LPS PC, we started by isolating RNA from the hippocampus of LPS-pretreated, vehicle, and control rats. Then, to examine the influence of LPS PC on specific genes expression, which may affect neuroprotection, we applied RT-PCR. Here, all results were normalized using the house-keeping gene GAPDH.

**FIGURE 6:**
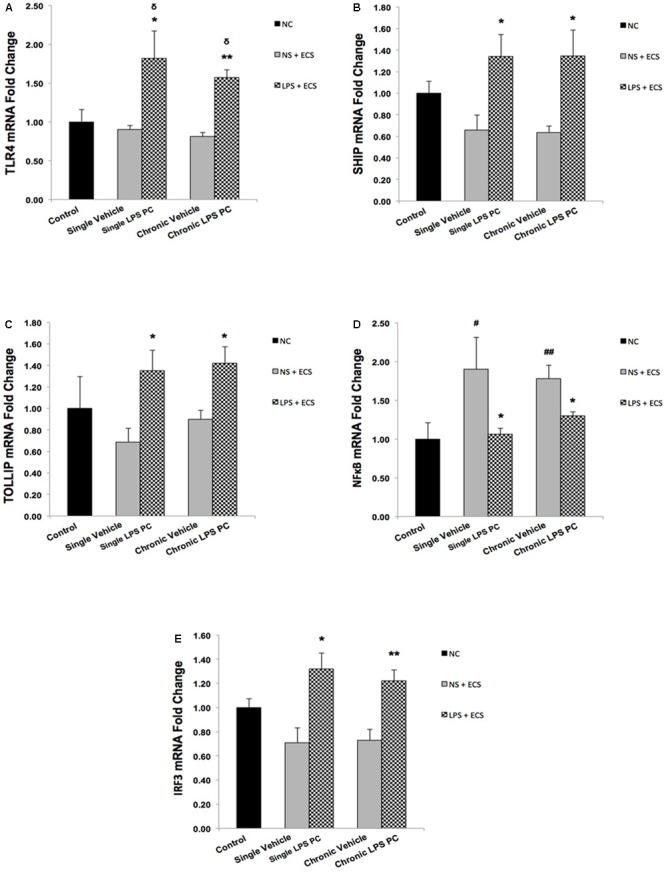
Expression level of genes related to TLR4 signaling pathway in the hippocampus region of rat brain in the ECS model of epilepsy. The graphs above display the effect of PC by single low dose and chronic ultra-low dose of LPS on the gene expression level of **(A)** TLR4, **(B)** SHIP1, **(C)** TOLLIP, **(D)** NFκB, and **(E)** IRF3 in hippocampus in control, single preconditioned, and chronic preconditioned groups. Asterisk (^∗^) denotes a significant difference between the preconditioned groups and vehicle groups. Delta (δ) denotes a significant difference between the preconditioned groups and control groups. Hash (#) denotes a significant difference between the vehicle groups and control groups. Statistical analysis has been done according to the one-way ANOVA with significance levels of ^∗^ρ < 0.05; ^∗∗^ρ < 0.01; ^δ^ρ < 0.05; ^#^ρ < 0.05; and ^##^ρ < 0.01. Data are shown as mean ± SEM of three animals (*n* = 3), which expressed as fold change relative to control group. ECS, electroconvulsive shock; NS, normal saline; LPS, lipopolysaccharide; PC, preconditioning; NC, no current was passed through the electrodes.

As shown in **Figure 6A**, we demonstrated that single and chronic PC showed significantly higher TLR4 gene expression in pretreated rats compared with the vehicle ones. Moreover, we also observed that PC with LPS resulted in a significant up-regulation in expression of SHIP1 (**Figure 6B**) and TOLLIP (**Figure 6C**) in single and chronic LPS-preconditioned groups compared with the vehicle animals. Following that, to determine the effect of LPS PC on inflammatory mediator production, NFκB expression level was also evaluated in preconditioned and vehicle groups. NFκB expression in the brain was elevated markedly in the vehicle groups; however, our study revealed that NFκB was significantly down-regulated in single and chronic preconditioned rats compared with the vehicle groups (**Figure 6D**). In addition, to determine the function of reprogrammed response after LPS PC, we compared the IRF3 gene expression level between preconditioned and vehicle groups. Our study demonstrated that IRF3 expression was significantly higher in the hippocampus of single and chronic LPS-preconditioned rats when compared to the vehicle animals (**Figure 6E**). Activation of IRF3 is associated with anti-inflammatory/type I IFN responses to enhance tolerance.

**FIGURE 7:**
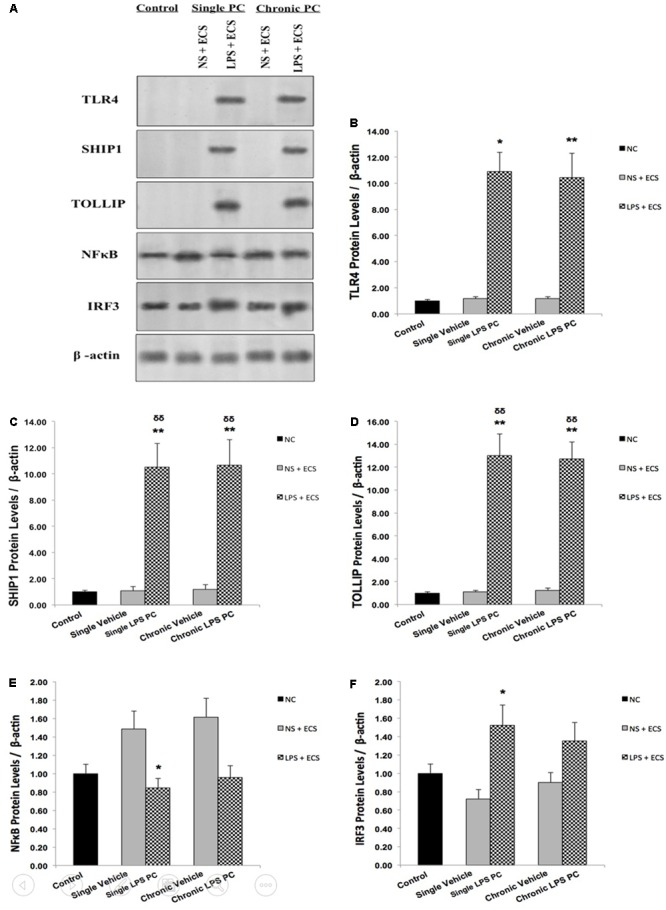
The level of protein expression in the hippocampus of rat brain in ECS model of Epilepsy. **(A)** Representative western blots in the ECS model of epilepsy showing protein expression of TLR4, SHIP1, TOLLIP, NFκB, and IRF3 in the hippocampus in control, single and chronic LPS-preconditioned, and vehicle groups. The graphs above display the effect of preconditioning by single low dose and chronic ultra-low dose of LPS on the protein expression level of **(B)** TLR4, **(C)** SHIP1, **(D)** TOLLIP, **(E)** NFκB, and **(F)** IRF3 in the hippocampus in control, single and chronic LPS-preconditioned, and vehicle groups. Asterisk (^∗^) denotes a significant difference between the preconditioned groups and vehicle groups. Delta (δ) denotes a significant difference between the preconditioned groups and control groups. Statistical analysis was done according to the one-way ANOVA with significance levels of ^∗^ρ < 0.05, ^∗∗^ρ < 0.01; and ^δδ^ρ < 0.01. Data are shown as mean ± SEM of three (*n* = 3), which expressed as fold change relative to control group. ECS, electroconvulsive shock; NS, normal saline; LPS, lipopolysaccharide; PC, preconditioning; NC, no current was passed through the electrodes.

In order to confirm the results obtained from real time PCR, we isolated total protein from hippocampal tissue of all animals including control, vehicle, and LPS-preconditioned rats and analyzed the protein expression of five biomarkers including TLR4, SHIP1, TOLLIP, NFκB, and IRF3 (**Figure 7A**). In this study, all results were normalized by β-actin as the reference protein. Western blot analysis revealed significant up-regulation in total protein levels of TLR4 in single and chronic LPS preconditioned rats when compared with vehicle animals (**Figure 7B**). To confirm whether LPS PC had induced new gene regulation in response to epileptic injury, the protein expression level of NFκB inhibitors was determined. The results from our experiment showed that the protein levels of SHIP1 and TOLLIP were up-regulated significantly in single and chronic LPS-preconditioned rats when compared with the vehicle groups (**Figures 7C,D**). In addition, PC with a single dose of LPS resulted in a significant reduction in NFκB protein expression in response to the ECS stimulus as compared to the vehicle groups, while, no significant difference in NFκB protein expression was observed between chronic LPS-preconditioned and vehicle groups (**Figure 7E**). To further explore whether LPS-induced protection is expressed through the TRIF pathway, the protein expression level of IRF3, a critical effector of tolerance, was also tested. Our study showed that single preconditioned rats displayed significantly higher protein expression of IRF3 when compared to the vehicle rats. However, there was no significant change in IRF3 protein expression in chronic preconditioned rats vs. vehicle animals (**Figure 7F**). Original western blots presented in Supplementary Figure 1.

### Effect of LPS Preconditioning on Anti-inflammatory Mediators

**FIGURE 8:**
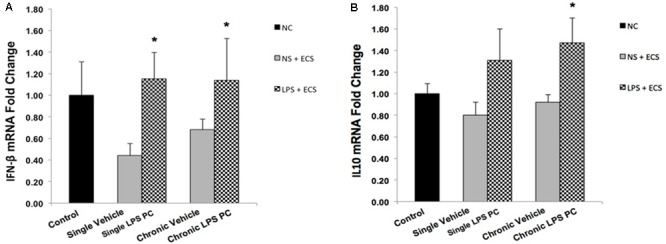
Gene expression level of IFN-β and IL10 in the hippocampus region of rat brain in the ECS model of epilepsy. The graphs above display the effect of preconditioning by single low dose and chronic ultra-low dose of LPS on the gene expression level of **(A)** IFN-β and **(B)** IL10 in hippocampus in control, single preconditioned, and chronic preconditioned groups. Asterisk (^∗^) denotes a significant difference between the preconditioned groups and vehicle groups. Statistical analysis has been done according to the one-way ANOVA with significance levels of ^∗^ρ < 0.05. Data are shown as mean ± SEM of three animals (*n* = 3), which expressed as fold change relative to control group. ECS, electroconvulsive shock; NS, normal saline; LPS, lipopolysaccharide; PC, preconditioning; NC, no current was passed through the electrodes.

To determine the protective effect of TLR4 activation by LPS PC against epileptic injury through the TRIF signaling pathway, we examined the expression level of anti-inflammatory mediators including IFN-β and IL10 in rat hippocampus. Rats preconditioned with single and chronic LPS stimulation resulted in a significant induction of RNA expression of IFN-β compared to the vehicle group (**Figure 8A**). Additionally, our study showed no significant change in the expression level of IL10 following single LPS PC vs. vehicle groups. However, significant increase in IL10 gene expression was observed in chronic LPS preconditioned rats (**Figure 8B**).

### Effect of LPS Preconditioning on Inflammatory Mediators

**FIGURE 9:**
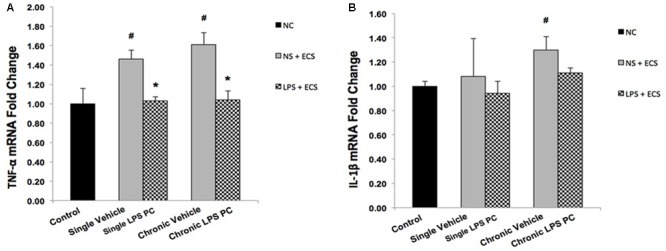
Gene expression level of TNF-α and IL-1β in the hippocampus region of rat brain in the ECS model of epilepsy. The graphs above display the effect of preconditioning by single low dose and chronic ultra-low dose of LPS on the gene expression level of **(A)** TNF-α and **(B)** IL-1β in hippocampus in control, single preconditioned, and chronic preconditioned groups. Asterisk (^∗^) denotes a significant difference between the preconditioned groups and vehicle groups. Hash (#) denotes a significant difference between the vehicle groups and control groups. Statistical analysis was done according to the one-way ANOVA with significance levels of ^∗^ρ < 0.05 and ^#^ρ < 0.05. Data are shown as mean ± SEM of three animals (*n* = 3), which expressed as fold change relative to control group. ECS, electroconvulsive shock; NS, normal saline; LPS, lipopolysaccharide; PC, preconditioning; NC, no current was passed through the electrodes.

Activity of NFκB is associated with inflammation in the brain, in response to damage by induction of pro-inflammatory mediators such as TNF-α and IL-1β. In this study, the contribution of LPS PC to alter expression levels of these pro-inflammatory markers after seizure induction has been demonstrated. Expression level of TNF-α in single and chronic LPS-pretreated rats were significantly reduced compared with vehicle groups (**Figure 9A**). As the results in **Figure 9B** revealed, no significant changes were observed in IL-1β gene expression level in single and chronic preconditioned rats compared with vehicle animals.

### Effect of LPS Preconditioning on Expression of Genes Related to Receptor Function Following Seizures

**FIGURE 10:**
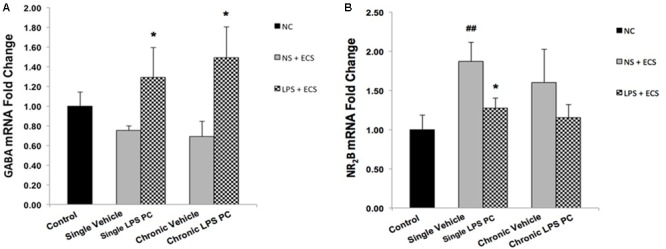
Gene expression level of GABA_A_ and NR_2_B in the hippocampus region of rat brain in the ECS model of epilepsy. The graphs above display the effect of preconditioning by single low dose and chronic ultra-low dose of LPS on the gene expression level of **(A)** GABA_A_ and **(B)** NR_2_B in hippocampus in control, single preconditioned, and chronic preconditioned groups. Asterisk (^∗^) denotes a significant difference between the preconditioned groups and vehicle groups. Hash (#) denotes a significant difference between the vehicle groups and control groups. Statistical analysis was done according to the one-way ANOVA with significance levels of ^∗^ρ < 0.05 and ^##^ρ < 0.01. Data are shown as mean ± SEM of three animals (*n* = 3), which expressed as fold change relative to control group. ECS, electroconvulsive shock; NS, normal saline; LPS, lipopolysaccharide; PC, preconditioning; NC, no current was passed through the electrodes.

To evaluate whether PC with LPS induced alterations in receptor function that are related to epilepsy, we examined gene expression levels of GABA_A_ and NR_2_B in preconditioned and vehicle groups. We found that preconditioned rats displayed a significant elevated expression in GABA_A_ compared to the vehicle animals (**Figure 10A**). In addition, single preconditioned LPS pretreated animals showed significantly lower levels of NR_2_B, compared to the vehicle group. However, the chronic LPS pretreated animals displayed no significant change in the level of NR_2_B in LPS pretreated animals compared to the vehicle group (**Figure 10B**).

## Discussion

Neuroinflammation and changes in levels of related pro-inflammatory cytokines lead to varying degrees of long-term alterations in the brain. Accumulating evidence strongly supports the relevance of neuroinflammation in the pathophysiology of epilepsy (Vezzani et al., 2013, 2016). Thus, novel therapeutic approaches should be investigated to effectively prevent or attenuate neuroinflammation related to epileptic seizures. In the current study, we have demonstrated that single and chronic brain LPS PC has important implications in terms of neuroprotection against epileptic injuries. Since previous studies indicated that hippocampal damage could produce behavioral impairments in animal epilepsy models (Dmowska et al., 2010), we performed Nissl staining following cell counting on hippocampal tissues collected from all animals. Histological analysis demonstrated that repeated ECS-induced tonic–clonic seizures were capable of producing cell loss in the hippocampal regions and these pathologic changes in the brain could cause behavioral impairments in the vehicle rats compared to preconditioned animals (**Figures 3–5**). These results lend support to the notion that behavioral changes induced by the administration of repeated ECS can be ascribed to cell loss in the hippocampal regions. In contrast, with reference to the results obtained from preconditioned animals, a sub-lethal dose of LPS was capable of inducing tolerance to alleviate cell death in the hippocampal regions and consequently improving behavioral impairments related to seizures. In both single and chronic LPS-pretreated animals, a significant reduction in seizure duration was observed when compared to the vehicle groups (**Figure 2**). This difference in seizure duration and also the attenuation of cell loss due to administration of ECS in the hippocampus could be attributed to the neuroprotective effect of LPS PC. These findings are in agreement with a prior study which reported that LPS PC could be protective and resulted in improvement of behavioral impairments as well as cell death (Dmowska et al., 2010). Thus, it is possible to conclude that single and chronic LPS PC may activate an endogenous protective mechanism that could influence animal behavior and histopathological changes.

As previous evidence suggested that inflammation could play a central role in epileptic disorders (Kosonowska et al., 2015; Vezzani et al., 2016), our findings further revealed that the inflammatory response following tonic–clonic seizures could be modulated by LPS PC. As such, we proceeded with molecular investigations to determine the effective role of LPS-induced tolerance wherein TLR4 signaling pathway reprograms in response to epileptic injury and directs it toward neuroprotection. Administration of low doses of LPS induces a mild inflammation in the brain that alters the response to subsequent damage and induces a new pattern of gene regulation in preconditioned animals. This alteration in response to injury is manifested by the reduction of inflammatory cascades and activation of anti-inflammatory mediators. To explore the reprogramming response-induced protection by brain LPS PC, we examined the expression levels of gene sets in response to epileptic injury. With regard to TLR4 signaling cascade as one the notable pathways related to LPS PC, our study found a significant increase in the expression of multiple markers including TLR4 (**Figure 6A**), SHIP1 (**Figure 6B**), TOLLIP (**Figure 6C**), and IRF3 (**Figure 6E**) in preconditioned animals compared with the vehicle groups; however, NFκB expression was significantly down-regulated (**Figure 6D**). These data that were obtained from gene expression were also confirmed by results from protein expression.

Activation and reprogramming of several signaling pathways in the brain in response to sub-lethal doses of LPS had been previously reported in patients with stroke (Vartanian et al., 2011; Wang et al., 2015). Activation of a set of genes that are evident in the reprogrammed response to damage such as SHIP1 and TOLLIP could negatively regulate the induction of pro-inflammatory mediators via inhibition of NFκB (Wang et al., 2015). Activation of NFκB participates in a chain of events that ultimately leads to the expression of a large number of pro-inflammatory cytokines such as TNF-α and IL-1β (Aalbers et al., 2014; Kołosowska et al., 2014). As demonstrated in our study, ECS-induced seizures in rat brain elicited over-expression of pro-inflammatory cytokines, while LPS PC mediated a significant reduction of TNF-α (**Figure 9A**). To support this, Rosenzweig et al. (2007) also reported that pretreatment with low doses of LPS diminished the production of pro-inflammatory mediators such as TNF-α and provided neuroprotection against cerebral ischaemic injury.

The mechanisms that underlie the neuroprotection afforded by LPS PC may also be involved in the augmentation of anti-inflammatory markers (Vartanian et al., 2011). In this regard, activation of IRF3 is required for LPS-induced neuroprotection through the production of anti-inflammatory mediators such as IFN-β (Marsh et al., 2009; Wang et al., 2015). In the current study, the RNA level of IFN-β and IL10 was markedly enhanced in response to seizures in preconditioned rats compared to the vehicle groups (**Figure 8**). Enhancement in gene expression levels of IFN-β in the LPS-preconditioned animals mirrors the cascade of protective processes that reduce inflammation in the brain (Marsh et al., 2009; Schaafsma et al., 2015).

**FIGURE 11:**
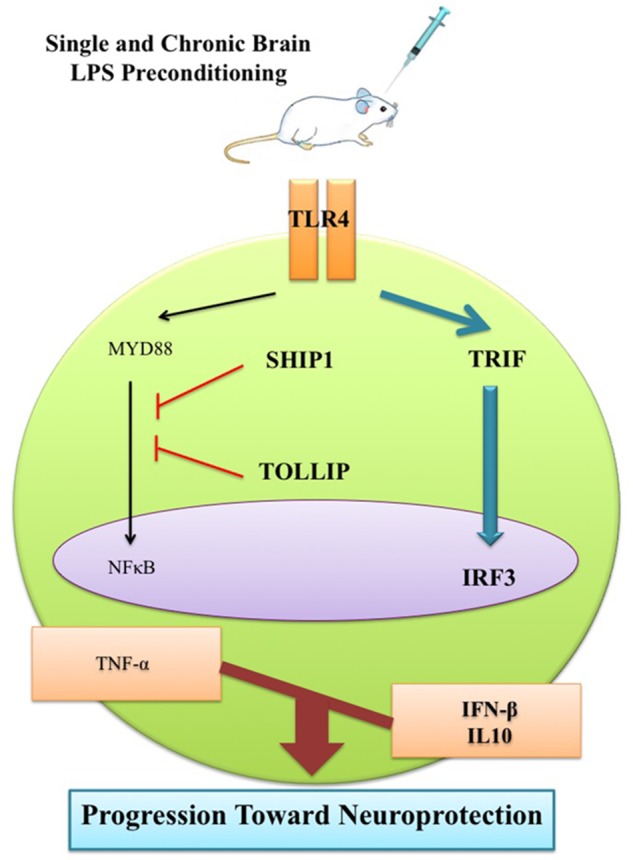
Schematic representation of gene expression pattern related to TLR4 signaling pathway following epileptic seizure. Low dose of LPS reprograms TLR4 signaling in response to subsequent brain injury. This reprogrammed response that is reminiscent of neuroprotection comes with reduction of NFκB activity to reduce pro-inflammatory mediators, up-regulation of IRF3 activity, and enhancement in production of anti-inflammatory/type I IFN associated genes in the LPS-preconditioned animals following brain damage.

This observation can be ascribed to the LPS-induced window of protection through the reprogramming of the response to epileptic injury via activation of TRIF-mediated signaling at the time of PC. Therefore, this supports the notion that single low dose and chronic ultra-low doses of LPS could potentially redirect the TLR4 signaling pathway and alter the endogenous response to injury resulting in a neuroprotective state via enhancement of anti-inflammatory mediators, reduction of pro-inflammatory cytokines, and finally modulation of inflammation in the brain (**Figure 11**). This shift from pro-inflammatory to anti-inflammatory response is considered a marker of reprogramming response in tolerance (Stevens et al., 2011; Ding et al., 2015).

Additionally, with respect to identifying the role of neurotransmitters in seizure mechanisms in the ECS model of epilepsy, the effect of LPS PC on gene regulation of two important receptors was investigated in all animal groups. Our study showed that GABA_A_ was significantly increased in LPS-preconditioned groups (**Figure 10A**), whereas NR_2_B gene expression was highly reduced in single preconditioned animals compared to the vehicle animals (**Figure 10B**). These results are in agreement with a previous study that demonstrated the capacity of PC to induce a neuroprotective response by decreasing glutamate release in neuron culture ischemia models (Tauskela et al., 2012). Therefore, our data and current medical literature support the role of LPS PC in neuroprotection. The role of neurotransmitters such as GABA_A_ and NR_2_B in the “neuroprotection cascade” provides insights to potential targets for new therapeutic approaches for epileptic seizures.

## Conclusion

Despite different administration of LPS doses (single and chronic), a similar reduction in neuroinflammation was observed. However, it is not clearly understood that there are some possibilities to explain this observation. These similarities that were observed between single and chronic administration of LPS could be due to the fact that both treatments may be reached at a ceiling effect for their dose. It is also possible that both treatments have the same efficacy at the doses used. Another possible reason for this may be due to that neuronal cells in hippocampus may become tolerant to subsequent exposures of LPS applied in chronic PC. In agreement with this, Schwarz et al. (2002) already reported a similar finding between acute and chronic treatment. In conclusion, the present findings have shed some light on the possible neuroprotective mechanisms of single low dose and chronic ultra-low doses of brain LPS PC observed in the ECS model of epilepsy. This tolerance was accompanied by a change in gene expression suggesting stimulation of a fundamental genomic reprogramming in the brain that confers survival.

## Author Contributions

AA designed the research. MG and EA performed behavioral, histological, and gene expression assessments and analyzed the data. AF and BK performed the protein assay. NI, AR, ZM, and AA supervised the project. All authors contributed to writing and editing the manuscript.

## Conflict of Interest Statement

The authors declare that the research was conducted in the absence of any commercial or financial relationships that could be construed as a potential conflict of interest.

## Abbreviations

β-Actin: beta actin
AP: anterior–posterior
BBB: blood–brain barrier
BSA: bovine serum albumin
CA1: Region 1 Cornu Ammonis
CA3: Region 3 Cornu Ammonis
cDNA: complementary DNA
CNS: central nervous system
DG: dentate gyrus
DV: dorso-ventral
ECS: electroconvulsive shock
GABA_A_ R: gamma-aminobutyric acid (GABA) type A receptor
GAPDH: glyceraldehyde 3-phosphate dehydrogenase
i.c.v: intracerebroventricular
i.p: intraperitoneal
IFC: integrated fluidic circuit
IFN-β: interferon-beta
IL10: interleukin 10
IL-1β: interleukin-1 beta
IRF3: interferon regulatory factor 3
LPS: lipopolysaccharide
ML: medio-lateral
mRNA: messenger RNA
MyD88: myeloid differentiation primary-response protein-88
n: number
NFκB: nuclear factor kappa-light-chain-enhancer of activated B cells
NMDAR: *N*-methyl D-aspartate receptor
NR_2_B: subunit-containing *N*-methyl-D-aspartate (NMDA) receptor
NS: normal saline
PC: preconditioning
PCR: polymerase chain reaction
RNA: ribonucleic acid
RT-PCR: real-time polymerase chain reaction
SD: Sprague Dawley
SEM: standard error of mean
SHIP1: Src homology 2-containing inositol phosphatase-1
Taq: *Thermus aquaticus*
TBST: Tris-buffered saline-Tween 20
TLR: Toll-like receptor
TNF-α: tumor necrosis factor-alpha
TOLLIP: Toll interacting protein
TRIF: TIR-domain-containing adapter-inducing interferon β
vs.: versus
