# Supplementary material for: Brain Lipopolysaccharide Preconditioning-Induced Gene Reprogramming Mediates a Tolerance State in Electroconvulsive Shock Model of Epilepsy
Source: Front Pharmacol. 2018 May 1;9:416. doi: 10.3389/fphar.2018.00416 (PMC5938816; doi:10.3389/fphar.2018.00416)
Supplement: Supplementary file 1 [file Image_1.PDF]

Supplementary Figure 1: Original uncropped images of western blots presented in Figure 7A.

Brain Lipopolysaccharide Preconditioning-Induced Gene Reprogramming Mediates a Tolerance State in Electroconvulsive Shock Model of Epilepsy

Elham Amini, Mojtaba Golpich, Abdoreza Soleimani Farjam, Behnam Kamalidehghan, Zahurin Mohamed, Norlinah Mohamed Ibrahim, Abolhassan Ahmadiani, Azman Ali Raymond

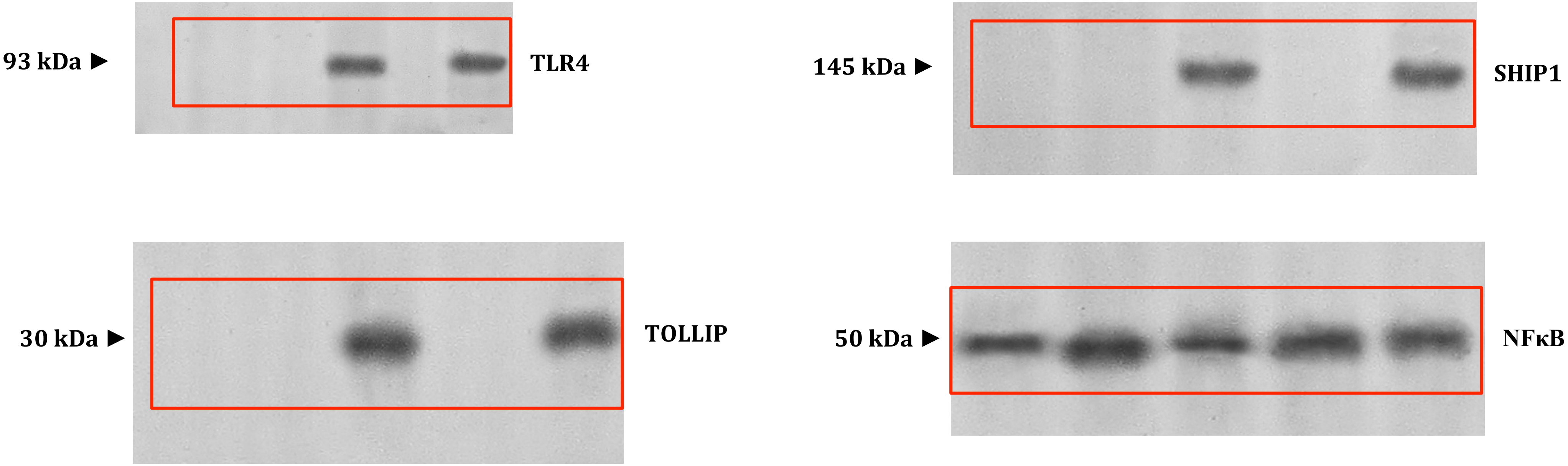

50 kDa ▶

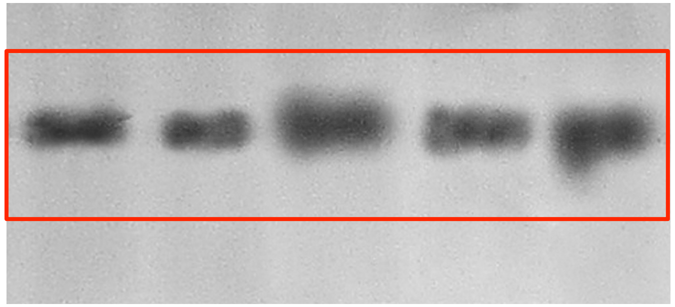

IRF3

42 kDa ▶

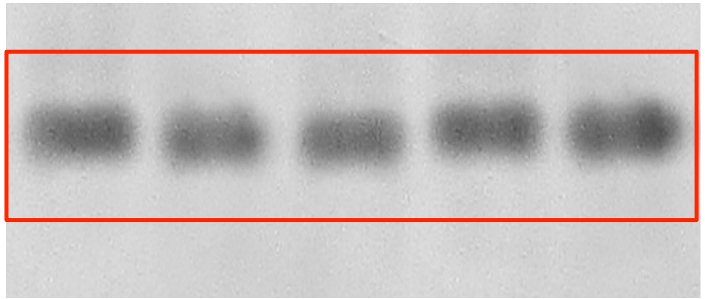

β-Acti

Figure 1. The corresponding main figure numbers are shown. Uncropped blots are the size of the membrane which was blotted. Membranes were cut according to the molecular weight and incubated with indicated antibodies. The red boxes indicate the cropped regions presented in main figures.
